# Who remains uncovered? Assessing inequalities and determinants of national health insurance enrolment among informal sector workers in Kenya

**DOI:** 10.1186/s41256-025-00461-7

**Published:** 2025-12-09

**Authors:** Phidelis Nasimiyu Wamalwa, Christoph Strupat, Kavita Singh, MaryBennah N. Kuloba, Jacob Kazungu, Manuela De Allegri

**Affiliations:** 1 Heidelberg Institute of Global Health, University Hospital and Medical Faculty, Heidelberg University, Im Neuenheimer Feld 130.3, 6th Floor, 69120 Heidelberg, Germany; 2 German Institute of Development and Sustainability (IDOS), Bonn, Germany; 3 Health Economics Research Unit, KEMRI Wellcome Trust Research Programme, Nairobi, Kenya; 4 Public Health Foundation of India, New Delhi, India

**Keywords:** National health insurance, Informal sector, Inequality, Universal health coverage, Enrolment, Missing-middle, Kenya

## Abstract

**Background:**

Many sub-Saharan African countries are increasingly adopting national health insurance policies to improve access to essential services. Informal sector workers, however, often lack coverage because their earnings are typically not low enough to qualify for government subsidies but insufficient to cover insurance premiums, resulting in a phenomenon known as "missing middle". This paper examined socioeconomic inequalities in national health insurance enrolment and determinants of participation among informal sector workers in Kenya.

**Methods:**

We used nationally representative cross-sectional household survey data (n = 5168) collected from informal sector workers in Kenya in December 2020. First, we examined levels of national health insurance enrolment among informal sector workers. Second, we examined socioeconomic inequalities in national health insurance enrolment using concentration curves and the Wagstaff index. Third, we employed a three-level mixed effects logistic regression model to assess the determinants of national health insurance enrolment.

**Results:**

Overall, 21.75% (95% Confidence Interval 20.63–22.89) of informal sector workers in Kenya were enrolled in the national health insurance scheme. We observed pro-rich inequalities in national health insurance enrolment, with a concentration index of 0.35 (95% CI 0.30–0.41). Older age (adjusted odds ratio (AOR) = 1.66, 95% CI 1.31–2.10), employment in the non-agricultural sector (AOR = 1.96, 95% CI 1.60–2.39), microfinance institutional membership (AOR = 1.44, 95% CI 1.23–1.69), higher education level (AOR = 2.49, 95% CI 1.99–3.11), household’s prior positive experience with healthcare (AOR = 1.45, 95% CI 1.22–1.72), and higher socioeconomic status based on the wealth asset index (AOR = 3.87, 95% CI 2.97–5.05) were all significantly positively associated with national health insurance enrolment. Larger households had lower odds of enrollment (AOR = 0.76, 95% CI 0.60–0.96).

**Conclusions:**

Our findings suggest that enrollment rates among informal sector workers remain low, and important pro-rich inequalities prevail. Economic factors, education, and prior experience with healthcare services were key drivers of national health insurance enrollment. Further policies are needed to increase enrollment among informal sector workers, including differential premium levels, reliance on expanded targeted subsidies, and enhanced awareness campaigns. Our findings are also applicable to other low-resource settings experiencing conditions similar to those in Kenya as they transition toward national health insurance policies, with the goal of achieving universal health coverage.

## Background

The United Nations Sustainable Development Goal 3, Target 3.8, aims to achieve universal health coverage (UHC) by 2030 [1]. Nevertheless, financing healthcare remains a challenge in low- and middle-income countries (LMICs) because of limited financial resources, competing demands, and slow economic growth [2, 3]. Over half of the world’s population lacks access to essential healthcare services, with one quarter facing financial hardship due to high out-of-pocket healthcare expenses [4]. This gap in healthcare access underscores the need for enhanced financial risk protection mechanisms, particularly among vulnerable populations. In sub-Saharan Africa (SSA), where a significant portion of the population lacks access to essential services [5–7], expanding social health protection mechanisms, such as health insurance, is crucial for achieving UHC targets. Although recommended, many LMICs face challenges in implementing fully tax-funded health financing systems due to limited public resources [8–10], especially in resource-constrained settings with large informal economies, where taxing a significant portion of the informal sector population is difficult [11, 12]. As a result, many countries that are also in the SSA region are increasingly adopting national health insurance (NHI) policies relying on hybrid models of tax-based subsidies and contributions raised either through payroll taxation or paid as direct income-adjusted premiums to foster progress toward UHC [13].

NHI is a form of publicly funded health insurance, an emerging health financing model designed to provide social health protection, regardless of individuals’ socioeconomic status (SES) [14, 15]. It leverages a co-funding model between the government and different population groups, in which contributions to the scheme are determined by the ability to pay. In contrast, benefits from the scheme should be determined by individual health needs [15, 16]. Under this model, vulnerable populations, such as the poor, are enrolled through direct government tax-subsidized premium contributions, whereas formal sector workers contribute via payroll taxes. [17–19]. However, the challenge arises for informal sector workers (ISWs), who are often expected to pay NHI premiums out-of-pocket. At the point of data collection, the monthly premium was a flat rate of Kenyan shillings (KES) 500 (~ United States Dollar (USD) 4.00) regardless of income, a premium that many struggle to afford, resulting in low enrolment and retention rates [9, 20, 21]. However, currently, it is based on 2.75% of the income or the value of the household assets determined by a means testing method, but not less than KES 300 (~ USD 3.75), a method that is likely to increase premiums for ISWs beyond what they can afford [22].

Despite many countries implementing laws and policies to include ISWs in NHI schemes, whether on a mandatory or voluntary basis, actual enrolment rates remain suboptimal, highlighting the gap between policy and practice [23]. These challenges have led many countries with established NHI schemes, such as Kenya [9, 20], Ghana [23, 24], Tanzania [25], and Zambia [26], to struggle with enforcing mandatory enrolment, resulting in low ISW coverage [13, 27, 28]. ISWs are generally characterized by low, unpredictable income [29, 30], limited awareness of enrolment processes and scheme benefits, and a lack of access to employer-based administrative structures [20, 31]. The COVID-19 pandemic has further impacted their economic status, leading to reduced incomes and livelihood loss, leaving many uninsured [32]. This has resulted in a phenomenon known as the "missing middle", in which a substantial segment of the population is left without health coverage, as their economic status disqualifies them from government subsidies but is not yet sufficient to cover insurance premiums [18], posing a critical barrier to increasing coverage and achieving equity [29, 30, 33].

The NHI scheme, the focus of this paper, is the main public health insurance provider established in 1966 as the National Hospital Insurance Fund. It initially provided mandatory coverage to formal sector workers through a compulsory employer deduction arrangement [34]. It was later expanded to include ISWs on a voluntary enrolment basis [35]. In December 2018, the Kenyan government launched a one-year "Afya Care" free healthcare policy pilot, which removed user fees in four counties, making health services free for all. The goal was to learn from the process and scale up the project to the rest of the country [36, 37]. However, considering government revenue volatility and the insufficient allocation of funding to the healthcare sector, a nationwide free healthcare system via taxation appeared unfeasible [38].

As part of the recent health financing reforms, Kenya, in 2020, adopted a mandatory enrolment policy for all population groups into the NHI scheme as a means toward achieving UHC [39]; however, as of 2022, only 24% of the population in Kenya was reported to be enrolled in the NHI scheme [40]. A few studies that have specifically examined ISWs in Kenya have reported that despite ISWs forming 84% of the employment sector [41], only 18% are enrolled in the NHI scheme [42]. Owing to their substantial population size, more ISWs need to be included in the NHI scheme to achieve UHC in Kenya, [43, 44]. Recent policy reforms, including the transition from voluntary to mandatory NHI enrollment, underscore the government’s commitment to expanding coverage beyond the formal sector. The availability of an ISW-specific nationally representative dataset during the transition period makes Kenya particularly an ideal setting for studying the real-world enablers and barriers to inclusive NHI enrolment, examining socioeconomic inequalities among ISWs, as well as offers timely and policy-relevant insights.

Understanding the socioeconomic inequalities and determinants of NHI enrolment among ISWs is, therefore, crucial for developing an effective strategy to implement the mandatory NHI policy. Despite this need, the available evidence on NHI enrolment among ISWs in Kenya is limited, with the few available studies having relatively small sample sizes and limited geographical and demographic diversity [8, 9]. Two studies used qualitative methods, which, while valuable, do not provide quantitative estimates [9, 31]. Furthermore, no study has examined the determinants of NHI enrolment in the complex post-pandemic period during which the transition from voluntary to mandatory enrolment occurred, and only a few studies have explored inequities in NHI enrolment, specifically among ISWs.

This study investigates socioeconomic inequalities and the determinants of NHI enrolment among ISWs in Kenya. By leveraging a large nationally representative dataset collected during the post-COVID-19 pandemic recovery period, our study fills a critical gap in the literature, focusing on a specific population group that is at the core of the challenge toward expanding social health protection programs in LMICs. Our work provides new empirical insights into persistent inequities and how structural and socioeconomic factors influence participation in NHI schemes in Kenya.

## Methods

### Study setting

Kenya is a lower-middle-income country with an estimated population of 47 million, 70% of whom are under 30 years of age [45], and a poverty rate of approximately 34% [46]. It is estimated that 84% of all employment opportunities are accounted for in the informal sector [42]. The Kenyan health system is organized around four hierarchical tiers based on the type of health services delivered: community (Level 1); primary care facilities (Levels 2–3); sub-county (primary) and county referral (secondary) hospitals (Levels 4–5); and national teaching and referral (tertiary) hospitals (level 6) [47]. The health sector is funded through government tax allocations, donor funding, and private sources, including out-of-pocket payments and private or public health insurance coverage [47]. The NHI scheme in Kenya covers a wide range of outpatient services at a specific health facility selected by the ISW household, as well as inpatient services in any accredited health facility in the country [48].

### Study design and population

We adopted an observational study design and conducted a cross-sectional analysis of a large, nationally representative survey exclusively among ISWs. The eligible population for the survey included informal sector workers aged 18 and above, defined as individuals employed in the informal economy sector, including those who were unemployed. The informal economy is defined as all economic activities by economic units that are, in law or practice, not covered or sufficiently covered by formal arrangements[49]. In Kenya, they include, but are not limited to, small-scale traders, farmers, domestic workers, individuals in the transport sector, and handicraft manufacturing [50].

The survey included a nationally representative sample of ISWs in Kenya. A clustered, stratified, multi-stage probability sample design ensured equal selection chances for all households operating in the informal economy, thereby providing a representative estimate of the views of the target population. This was achieved by strictly applying random selection methods at each sampling stage and sampling with a probability proportionate to the adult population size. The sampling process involved stratifying the country into regions, regions into counties, and counties into districts, which were further divided into villages. The primary sampling units or clusters were then allocated from each stratum based on the share of the national population and further allocated according to the urban–rural divide. A paper by Strupat [51] provides a detailed description of the sampling design, inclusion and exclusion criteria, field procedures, and processes.

### Data source and collection procedures

We used a secondary dataset collected in December 2020 by the German Institute of Development and Sustainability (IDOS) in collaboration with the Friedrich-Ebert-Stiftung (FES), the International Labor Organization (ILO), and the Institute for Development Studies at the University of Nairobi. The survey was conducted within the framework of a larger study aimed at evaluating social protection and social cohesion in the informal sector. For our research, we focused only on NHI participation as a relevant social protection outcome and extracted exclusively relevant information from the dataset. In-person interviews were conducted with the household head, who provided information about all the household members. A total of 2,608 households were sampled for interviews, representing 9,682 individuals. Considering the applicable legal age to enroll in the NHI scheme voluntarily, we only included in our analysis individuals aged 18 years and older, totaling 5,168.

### Variables

Table 1 below describes all the study variables, their definitions, and their distributions in the sample. The outcome variable, NHI enrolment, is dichotomous, indicating whether an individual is enrolled in the NHI scheme (1) or not (0). We included twelve explanatory variables grouped into individual, household head, and household-level characteristics. These were selected based on available data and informed by previous studies to facilitate a comparison of results [24, 52–56]. The individual characteristics included age, sex, and employment sector. The household head characteristics included education level [55]. The household-level characteristics included location, membership in microfinance institutions (MFIs)[57],^1^ household size, exposure to free health policy, wealth quintiles, any reported illness in the household, and experience with care of any household member (composite variable based on last health facility visit experience on four aspects: reception at the facility, competency of medical staff, ease of accessing the service they needed, and waiting time). We used a household asset-based index to compute wealth quintiles as a proxy for individual wealth (SES), as asset ownership fluctuates less than income or expenditures do [58].

**Table 1:** Definition and general distribution of the study variables (N = 5,168)

Variable	Definition	Measurement	Sample distribution % (n)
Outcome variable			
NHI enrolment	Individual NHI enrolment status	0 = No 1 = Yes	78.25 (4044) 21.75 (1124)
Individual characteristics			
Age	Individual age in years	1 = 18–34 years 2 = 35–54 years 3 = > 54 years	53.98 (2790) 32.51 (1680) 13.51 (698)
Sex	Individual sex	0 = Male 1 = Female	48.43 (2503) 51.57 (2665)
Employment sector	Individual employment sector	1 = Unemployed 2 = Agricultural sector 3 = Non-agricultural sector	28.93 (1403) 26.91 (1305) 44.16 (2142)
Household head characteristics			
Education	Household head’s educational level	1 = No basic education 2 = Primary education 3 = Secondary or higher-level education	31.49 (1627) 34.11 (1763) 34.40 (1778)
Household characteristics			
MFI Membership	Any eligible individual in the household reporting membership in a microfinance institution	0 = No 1 = Yes	66.74 (3449) 33.26 (1719)
Household size	Total number of members in a household	1 = Small < 4 2 = Average 4–6 3 = Large > 6	26.04 (1346) 46.85 (2421) 27.11 (1401)
Location	Household location	1 = Urban 2 = Rural	30.50 (1576) 69.50 (3592)
‘Afya Care’ free health care policy	Household located in one of the free healthcare pilot counties (in 2019)	0 = Yes 1 = No	93.21 (4817) 6.79 (351)
Wealth quintiles	Wealth composite index	1 = Poorest 2 = Poor 3 = Middle 4 = Rich 5 = Richest	20.34 (1051) 20.20 (1044) 19.97 (1032) 20.16 (1042) 19.33 (999)
Household experience with healthcare service delivery	Any household member’s experience with healthcare service delivery	0 = Unsatisfied 1 = satisfied	28.37 (1466) 71.63 (3702)
Any reported illness in the household	At least one household member reported being ill in the prior 12 months	0 = No 1 = Yes	59.71 (3086) 40.29 (2082)

### Statistical analysis

We first described the characteristics of our study sample and computed proportions of NHI enrolment using univariate and bivariate descriptive analysis. We then used Pearson’s chi-square test (χ^2^) to test the associations between the NHI score and explanatory variables. Second, we examined the presence of socioeconomic inequalities in NHI scheme enrolment, beginning with a simple comparative analysis using absolute differences and the high-to-low ratio as a measure for relative difference. We further analysed the inequalities using concentration curves (CC) and Wagstaff concentration index (CIX). The absolute difference was calculated as the arithmetic difference in the NHI enrolment rates between subgroup categories (category minus reference category), providing a measure of the magnitude of inequality in percentage points. For variables with more than two subgroups, we calculated pairwise comparisons of inequality for each category with the first or lowest category as the reference category. Similarly, we computed the relative ratio by dividing the insurance enrollment rate of the other categories by the reference (first/lowest) category, resulting in a relative measure of disparity. These measures were chosen as they provide a straightforward gradient of inequality in a simple way [59].

The CC plots the cumulative percentage of the variable of interest (NHI scheme enrolment) on the y-axis against the cumulative proportion of the population on the x-axis, ranked by SES from the poorest to the richest [58]. The 45° line indicates the line of equality, from which a deviation suggests the presence of SES inequality. A curve below (above) the equality line indicates a pro-rich (poor) distribution of enrolment [58]. We used the Wagstaff CIX to measure the magnitude of the inequality, defined as twice the area between the CC and the line of equality. The index’s values range from − 1 to + 1, with values closer to + 1 indicating a stronger concentration among the rich, while a negative value indicates a pro-poor distribution. A CIX of zero denotes equal distribution [58]. The CIX calculation was based on Wagstaff’s normalized CIX, an adjusted version of the standard CIX used when a variable of interest is a dichotomous variable with a lower bound of 0 and an upper bound of 1 [60]. The adjustment rescales the index of bounded variables so that it ranges from -1 to + 1, allowing for comparison across groups [60]. To comprehensively understand the sources of inequality, we further generated concentration curves and indices for individual explanatory variables.

Third, we used a three-level multilevel mixed effects logistic regression to analyse data from 5168 individuals nested within 47 counties (clusters). Owing to the hierarchical nature of the data, the model we employed accommodates both random and fixed effects, accounting for variability between clusters. We fitted four mixed-effect models to determine the best fit for the data. To determine which variable to include in the model, we conducted a bivariate unadjusted analysis, and all variables found to be significant at P < 0.05 were incorporated into the regression model. Although sex was not significantly associated, we included it in the model based on the current literature review. The initial model (M0) was null or intercept-only, fitted without independent variables. Model I (M1) was fitted with individual-level characteristics added to M0. Model II (M2) included household head and household level characteristics in addition to M0, while Model III (M3—full model) combines all individual and household-level characteristics. The Stata command "melogit" was used to fit these models based on the following formula:

$$\log it\left( {P\left( {Y_{ij} \, = \,1} \right)} \right)\, = \,\beta_{0} \, + \,\beta_{1} X_{ij} \, + \, \ldots \, \beta_{n} X_{nij \, + } u_{j}$$

where Y_ij_ is our binary outcome of the *i*th individual in the *j*th group (county clusters) being enrolled in the NHI scheme (Y_ij_ = 1), whereas Y_ij_ = 0 denotes the individual who is not enrolled. logit(P(Y_ij_ = 1)) is the log odds for Y_ij_ = 1, and ß_0_ is the fixed intercept. β_1…_ β_n_ are the fixed effect coefficients for explanatory variables X_ij…_ X_nij_; *u*_j_ is the random effect for group j, often modelled as u_j ~N(0,_ σ^2^).

We used adjusted odds ratios (AORs) with 95% confidence intervals (CIs) to analyse and present fixed effects. We used the intraclass correlation coefficient (ICC) and proportional change in variance (PCV) to check the random variation across clusters. Model selection was based on Akaike’s Information Criterion (AIC), which favors the model with the lowest value. Multicollinearity was assessed using the variance inflation factor (VIF), with all variables showing low VIF values < 2 [61], resulting in an overall VIF of 1.41.

## Results

Our analysis included a total of 5168 ISWs aged 18 years and above. Among these, more than half (53.99%) were aged 18–34 years, with a slightly greater percentage of females (51.57%) than males (48.53%). Approximately two-thirds (69.50%) lived in rural areas, while one-third (30.50%) had no formal education. Overall, the NHI scheme enrolment rate among ISWs was 21.75% (95% CI 20.63–22.89) (Table 1). Table 2 presents the comparison of the distribution of each categorical variable between the insured and uninsured ISWs. Insured members were more likely to come from wealthier households, households with more educated heads, be employed in the non-agricultural sector, have at least one member with MFI membership within the household, and those exposed to the UHC pilot (p-value < 0.001). Our analysis revealed no significant association between sex and NHI enrolment.

**Table 2:** Characteristics of the study population insured and not insured in the NHI scheme

Variables	Total (N = 5168)	Insured (N = 1124)	Uninsured (N = 4044)	P value
Continuous Age Household size		Mean (95% CI)	Mean (95% CI)	
	38.2 (35.72–36.54) 5.13 (5.07–5.19)	38.2 (37.4–39.0) 4.7 (4.6–4.8)	35.6 (35.1–36.0) 5.2 (5.1–5.3)	< 0.001 < 0.001
Categorical	% (n)	% (n)	% (n)	
Individual characteristics				
Age 18–34 years 35–54 years > 54 years	53.99 (2790) 32.51 (1680) 13.51 (698)	47.24 (531) 38.08 (428) 14.68 (165)	55.86 (2259) 30.96 (1252) 13.18 (533)	< 0.001
Sex Male Female	48.43 (2503) 51.57 (2665)	48.75 (548) 51.25 (576)	48.34 (1955) 51.66 (2089)	0.807
Employment sector Unemployed Agricultural Non-Agricultural	28.93 (1403) 26.91 (1305) 44.16 (2142)	19.19 (209) 22.96 (250) 57.85 (630)	31.75 (1194) 28.05 (1055) 40.20 (1512)	< 0.001
Household head characteristics				
Education No schooling Primary Secondary and higher-level	31.48 (1627) 34.11 (1763) 34.40 (1778)	16.55 (186) 27.85 (313) 55.60 (625)	35.63 (1441) 35.86 (1450) 28.51 (1153)	< 0.001
Household-level characteristics				
Household size < 4 4–6 > 6	26.04 (1348) 46.85 (2421) 27.11 (1401)	31.85 (358) 50.71 (570) 17.44 (196)	24.43 (988) 45.77 (1851) 29.80 (1205)	< 0.001
MFI membership No Yes	66.74 (3449) 33.26 (1719)	56.49 (635) 43.51 (489)	69.58 (2814) 30.42 (1230)	< 0.001
Location Rural Urban	69.50 (3592) 30.50 (1576)	61.21 (688) 38.79 (436)	71.81(2904) 28.19 (1140)	< 0.001
‘Afya Care’ Free Health Care Policy Pilot Nonpilot	6.79 (351) 93.21 (4817)	9.70 (109) 90.30 (1015)	5.98 (242) 94.02 (3802)	< 0.001
Wealth quintiles Poorest Poor Middle Rich Richest	20.34 (1051) 20.20 (1044) 19.97 (1032) 20.16 (1042) 19.33 (999)	9.26 (104) 13.61 (153) 15.57 (175) 23.84 (268) 37.72 (424)	23.42 (947) 22.03 (891) 21.19 (857) 19.14 (774) 14.22 (575)	< 0.001
Household experience with healthcare service delivery Good Bad	71.63 (3702) 28.37 (1466)	77.31 (869) 22.69 (255)	70.05 (2833) 29.95 (1211)	< 0.001
Any reported illness in the household No Yes	59.71 (3086) 40.29 (2082)	64.68 (727) 35.32 (397)	58.33 (2359) 41.67 (1685)	< 0.001

N = 5168; NHI = National health insurance; CI = 95% Confidence Interval; n = group categories subsample; p value = calculated using Pearson’s chi^2^ test at 95% CI

Table 3 presents the absolute and relative differences, as well as category-specific CIX, for NHI enrollment among ISWs. ISWs in the richest and rich quintiles were 4 and 2 times more likely to enrol compared to those in the poorest quintile (relative difference = 4.29 and 2.60, respectively). Marked disparities were observed across education levels, with an absolute difference of 23.72 percentage points and a relative ratio of 3.08. The highest education group was 3 times more likely to be enrolled than those with no basic education. Our results show an absolute difference of 14.51, a relative ratio of 1.97, and a positive, significant CIX in NHI coverage between ISWs employed in the non-agricultural sector and the unemployed. This reveals a sizeable gap in NHI enrolment, with the ISWs employed in the non-agricultural sector nearly two times more likely to be enrolled compared to the unemployed group, indicating a pro-rich inequality. NHI enrolment was significantly lower in large (> 6 members) households compared to the small (< 4 members) households. An absolute difference of -12.61 and a relative ratio of 0.53 indicate that ISWs in large households were less likely to enrol than those from smaller households, with the CIX still showing concentration among the pro-rich.

**Table 3:** Equity results for absolute differences, relative differences, and CIX (N = 5168)

Variables	Insured% % (n)	Absolute difference	Relative Ratio	CIX	Robust Standard Error of CIX
NHI scheme		-	-	0.35	0.03***
Age 18–34 years (Ref) 35–54 years > 54 years	19.03 (531) 25.48 (428) 23.64 (165)	6.45 4.61	1.34 1.24	0.30 0.39 0.45	0.03*** 0.05*** 0.07***
Sex Male (Ref) Female	21.89 (548) 21.61 (576)	-0.28	0.99	0.33 0.41	0.03*** 0.03***
Employment sector Unemployed (Ref) Agricultural Non-Agricultural	14.90 (209) 19.16 (250) 29.41 (630)	4.26 14.51	1.29 1.97	0.34 0.36 0.38	0.05*** 0.06*** 0.03***
Education No schooling (Ref) Primary Secondary and higher-level	11.43 (186) 17.75 (313) 35.15 (625)	6.32 23.72	1.55 3.08	0.28 0.24 0.29	0.06*** 0.06*** 0.04***
Household size < 4 (Ref) 4–6 > 6	26.60 (358) 23.54 (570) 13.99 (196)	-3.06 -12.61	0.53 0.88	0.41 0.36 0.32	0.05*** 0.04*** 0.06***
Location Rural (Ref) Urban	19.15 (688) 27.66 (436)	8.51	1.44	0.36 0.36	0.04*** 0.05***
MFI membership No (Ref) Yes	18.41 (635) 28.45 (489)	10.04	1.55	0.35 0.34	0.04*** 0.04***
Wealth quintiles Poorest (Ref) Poor Middle Rich Richest	9.90 (104) 14.66 (153) 16.96 (175) 25.72 (268) 42.44 (424)	4.76 7.06 15.82 32.54	1.48 1.71 2.60 4.29	- - - - -	- - - - -
‘Afya Care’ free health care policy Nonpilot (Ref) Pilot	21.07 (1015) 31.05 (109)	9.98	1.47	0.38 0.20	0.03*** 0.19
Household experience with healthcare service delivery Bad (Ref) Good	17.39 (255) 23.47 (869)	6.08	1.35	0.30 0.40	0.07*** 0.04***
Any reported illness in the household No (Ref) Yes	23.56 (727) 19.07 (397	-4.49	0.81	0.38 0.35	0.04*** 0.05***

CIX = Concentration Index; 95% significance levels * p < 0.1, ** p < 0.05, ***p < 0.01; Ref-Reference category

**Fig. 1:**
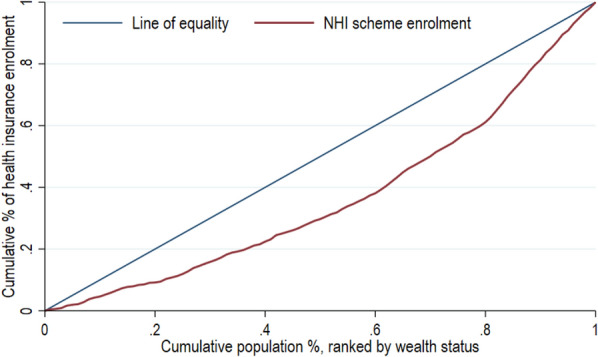
Concentration curve for ISW’s enrolled in the NHI scheme

Our inequality analysis reveals a moderate pro-rich distribution of NHI enrolment among ISWs. The CC in Fig. 1 illustrates that enrollment in the NHI is mostly concentrated among the wealthiest ISWs, as confirmed by the CIX estimation (Table 3), which is estimated at a value of 0.35 (95% CI 0.30–0.41; p-value < 0.001).

**Fig. 2:**
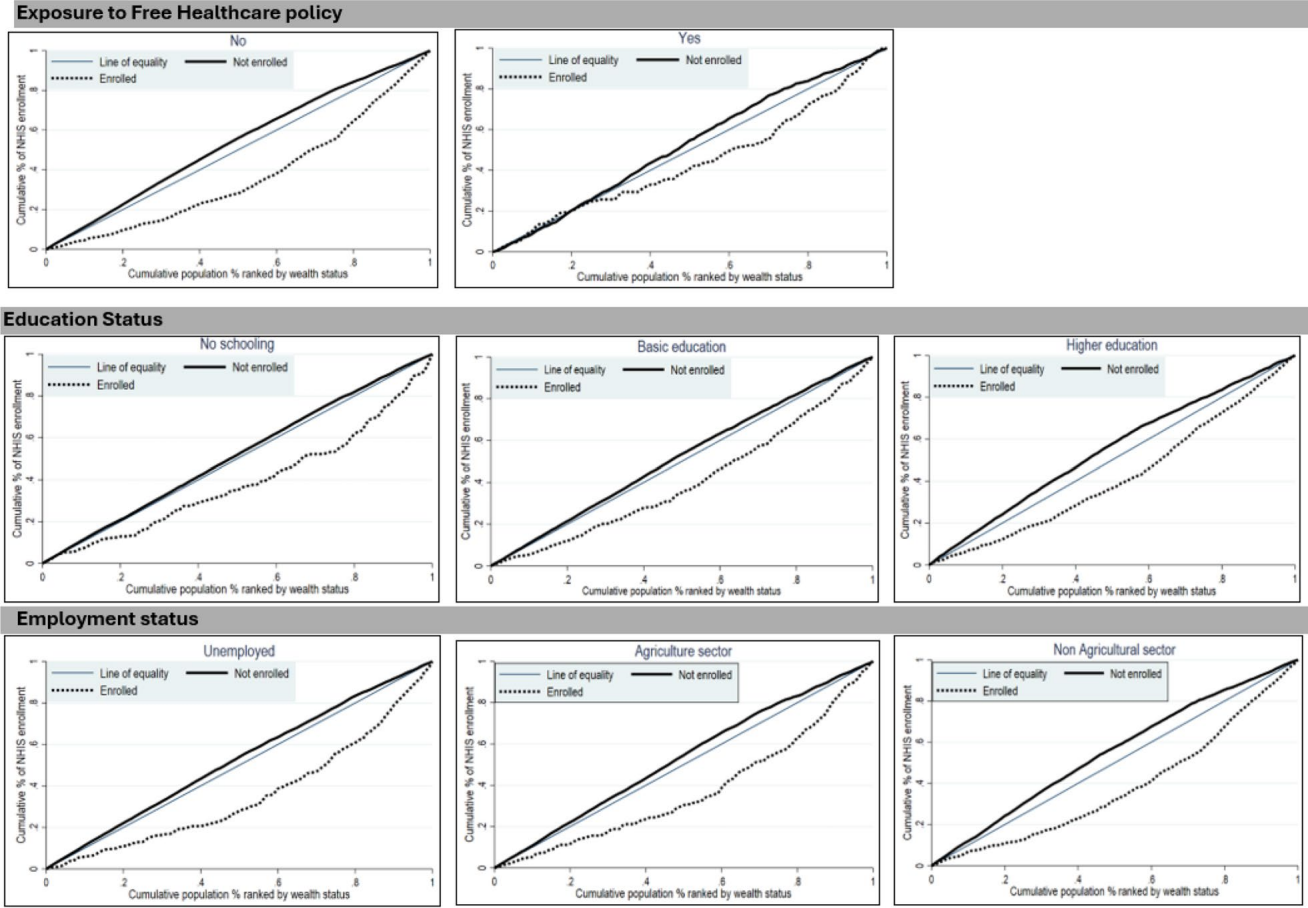
Concentration curves to compare NHI enrolment SES inequity based on exposure to free healthcare policy, education, and employment status

Figure 2 shows CCs comparing NHI enrolment among ISWs based on exposure to free healthcare policy, education, and employment status, with accompanying CIX presented in Table 3. NHI enrolment was more equitable among the poorest ISWs (lowest 20%) exposed to the free healthcare policy pilot than among those not exposed, with the curve for ISWs exposed to the policy even slightly crossing the line of equality for the poorest quintile. This effect was, however, not significant, as the other quintiles remained below the line of equality (CIX = 0.20, p-value = 0.341). Although the household heads’ education level had the least pro-rich inequality based on the CIX, the CC showed that even among wealthier quintiles, ISWs belonging to household heads with no basic education, the unemployed, and those employed in the agricultural sector were far less likely to enrol compared to those with basic or advanced education levels.

As shown in Table 4, based on the random effect analysis results, the null model ICC indicates that a significant 13% of the variation in NHI enrolment is attributable to differences among clusters. The PCV value indicates that the combined effect of individual and household variables accounts for 29% of the variation in NHI enrollment among ISWs. Model IV (Full Model) had the lowest AIC value (4488), indicating that it is the best-fit model.

**Table 4:** Multilevel mixed effect model output for determinants of health insurance status (full model, N = 5168)

Variable	Model 0 (Null)	Model 1 AOR (95% CI)	Model 2 AOR (95% CI)	Model 3 AOR (95% CI)	Model 4 (Full) AOR (95% CI)
Model Parameters					
Variance	0.48	0.49	0.38	0.29	0.34
Intraclass correlation coefficient (ICC)	0.13	0.13	0.10	0.08	0.09
Proportion of Change in Variance (PCV) %	Reference	2.08	26	40	29
Model fitness					
AIC	5184.94	4860.09	4946.17	4882.27	4488.32
BIC	5198.04	4905.49	4978.92	4960.87	4618.05
Individual Characteristics					
Age in years 18–34 35–54 > 54		1.00 1.31 (1.11–1.53) *** 1.46 (1.17–1.83) ***			1.00 1.35 (1.13–1.60) ** 1.63 (1.29–2.07) ***
Sex Male Female		1.04 (0.90–1.20)			1.00 1.05 (0.90 -1.22)
Employment sector Unemployed Agricultural Non-Agricultural		1.00 1.09 (0.87–1.37) 2.13 (1.76–2.57) ***			1.00 1.16 (0.92 -1.48) 1.96 (1.61–2.40) ***
Household head characteristics					
Highest education level No schooling Primary Post-primary			1.00 1.48 (1.20–1.83) *** 3.54 (2.89–4.32) ***		1.00 1.24 (0.99–1.55) * 2.52 (2.01–3.15) ***
Household characteristics					
Location Rural Urban				1.00 1.15 (0.93–1.42)	1.00 1.05 (0.84–1.32)
‘Afya Care’ free health care policy Unexposed Exposed				1.00 1.95 (1.03–3.68) **	1.00 1.75 (0.88–3.46)
MFI membership No Yes				1.00 1.59 (1.37–1.85) ***	1.00 1.41 (1.20–1.66) ***
Household size < 4 4–6 > 6				1.00 0.91 (0.77–1.07) 0.56 (0.45.0.70) ***	1.00 1.00 (0.84–1.20) 0.73 (0.57–0.92) **
Wealth quintiles Poorest Poor Middle Rich Richest				1.00 1.30 (0.99–1.71) * 1.96 (1.52–2.52) *** 2.78 (2.17–3.56) *** 5.66 (4.43–7.23) ***	1.00 1.25 (0.95–1.66) 1.70 (1.30–2.21) *** 2.19 (1.68–2.85) *** 3.87 (2.97–5.05) ***
Household experience with healthcare service delivery Unsatisfied Satisfied				1.00 1.45 (1.23–1.71) ***	1.00 1.49 (1.22–1.73) ***
Any reported illness in the household No Yes				1.00 0.96 (0.83–1.11)	1.00 0.88 (0.84—1.15)

Total sample = 5168; 95% significance levels * p < 0.1, ** p < 0.05, ***p < 0.01; AOR-adjusted odds ratio; CI-Confidence Intervala

Table 4 displays the results for the best-fit multilevel mixed-effect logistic regression model (Model 3). The findings show that among the individual variables, age and employment status were significantly associated with NHI enrolment. Compared with ISWs aged 18–34 years, those aged 34–54 years and 54 + years were 1.35 times (AOR 1.35; 95% CI 1.13–1.60) and 1.63 times (AOR 1.63; 95% CI 1.29–2.07) more likely, respectively, to enrol in the NHI scheme. Compared with those who were unemployed, ISWs employed in the non-agricultural sector had twice the odds of enrolling in the NHI scheme (AOR = 1.96; 95% CI 1.61–2.40). Sex (AOR = 1.05; 95% CI 0.90–1.22) was not associated with NHI scheme enrolment. From the perspective of household head characteristics, compared with those with no formal education, ISWs in households whose heads had advanced education levels (tertiary or university) had twice the odds of enrolling in the NHI scheme(AOR 2.52; 95% CI 2.01–3.15) (Table 5).

**Table 5:** Multilevel mixed-effect logistic regression analysis model output for determinants of health insurance status

Variable	Bivariate analysis OR (95% CI)	Model 3 AOR (95% CI)	Predicted probabilities for HI status -% (95% CI)
Individual Characteristics			
Age in years 18–34 35–54 > 54	1.00 1.51 (1.30–1.75) *** 1.47 (1.19–1.82) ***	1.00 1.35 (1.13–1.60) ** 1.63 (1.29–2.07) ***	20.52 (17.65–23.39) 24.83 (21.46–28.20) 27.86 (23.43–32.30)
Sex Male Female	0.96 (0.84–1.10)	1.00 1.05 (0.90 -1.22)	22.64 (19.63–25.65) 23.32 (20.26–26.38)
Employment sector Unemployed Agricultural Non-Agricultural	1.24 (1.00–1.54) ** 2.26 (1.88–2.71) ***	1.00 1.16 (0.92 -1.48) 1.96 (1.61–2.40) ***	17.84 (14.76–20.93) 19.82 (16.58–23.05) 27.70 (24.22–31.18)
Household head characteristics			
Education No schooling Completed Primary Post primary	1.52 (1.24–1.88) *** 3.65 (1.99–4.46) ***	1.00 1.24 (0.99–1.55) * 2.52 (2.01–3.15) ***	16.64 (13.54–19.75) 19.43 (16.35–22.52) 30.73 (26.86–34.61)
Household characteristics			
Location Rural Urban	1.41 (1.15–1.73) **	1.00 1.05 (0.84–1.32)	22.73 (19.86–25.60) 23.47 (19.62–27.31)
MFI membership No Yes	1.67 (1.44–1.94) ***	1.00 1.41 (1.20–1.66) ***	21.08 (18.23–23.94) 26.25 (22.78–29.71)
‘Afya Care’ FHC policy Unexposed Exposed	2.14 (1.02–4.50) **	1.00 1.75 (0.88–3.46)	22.34 (19.41–25.26) 31.24 (19.93–42.55)
Household size < 4 4–6 > 6	0.94 (0.80–1.11) 0.60 (0.49–0.75) ***	1.00 1.00 (0.84–1.20) 0.73 (0.57–0.92) **	24.02 (20.54–27.50) 24.03 (20.89–27.17) 19.51 (16.19–22.83)
Wealth quintiles Poorest Poor Middle Wealthy Wealthiest	1.29 (0.98–1.68) * 1.86 (1.44–2.39) *** 2.70 (2.10–3.45) *** 5.54 (4.34–7.10) ***	1.00 1.25 (0.95–1.66) 1.70 (1.30–2.21) *** 2.19 (1.68–2.85) *** 3.87 (2.97–5.05) ***	14.51 (11.47–17.56) 17.22 (13.77–20.67) 21.40 (17.70–25.10) 25.40 (21.42–29.38) 35.81 (31.16–40.46)
Household experience with healthcare service delivery Bad Good	1.43 (1.22–1.68) ***	1.00 1.49 (1.22–1.73) ***	19.00 (15.97–22.03) 24.62 (21.59–27.66)
Any reported illness in the household No Yes	0.87 (0.75–0.99) **	1.00 0.88 (0.84—1.15)	23.09 (20.05–26.15) 22.87 (19.82–25.92)

Total sample = 4850; 95% significance levels * p < 0.1, ** p < 0.05, ***p < 0.01; AOR-adjusted odds ratio

As shown in Table 5, among the household characteristics, wealth status, household size, and experience with healthcare services received were significantly associated with enrollment in the NHI scheme. Compared with the poorest quintile, ISWs in the wealthy and wealthiest quintiles were two times (AOR = 2.19; 95% CI 1.68–2.85) and three times (AOR = 3.87; 95% CI: 2.97–5.05) more likely to enrol in the NHI scheme. The odds of enrolling in the NHI scheme were 41% higher among ISWs with informal MFI membership (AOR = 1.41; 95% CI: 1.20–1.66) than among those with no MFI membership. ISWs from households that had at least one person reporting a positive experience in their last visit to a health facility were 1.49 times more likely to enrol than those from a household that had any individual reporting a bad experience (AOR 1.49; CI: 1.22–1.73). Additionally, ISWs from large households (> 6 members) were 73% less likely to enrol (AOR = 0.73; 95% CI: 0.57–0.92). Despite having a large effect size (AOR = 1.75; 95% CI: 0.88–3.46), exposure to free healthcare policy was not significantly associated with NHI scheme enrolment. Similarly, the location of residence and self-reported illness were not associated with enrollment in the NHI scheme.

## Discussion

This paper makes an important contribution to the health insurance literature by focusing on determinants of NHI enrolment among ISWs. This population segment remains challenging to include in NHI schemes but is critical for achieving UHC. The reliance on nationally representative survey data that specifically targets ISWs makes the study findings unique and represents one of the first national household-level studies to examine NHI penetration levels and determinants of enrolment in the NHI among ISWs in Kenya. Furthermore, the study adds valuable insights to the literature regarding inequality analysis among ISWs in this LMIC context. Timing is also essential since survey data were collected post-pandemic, a crisis that could have impacted participation in the scheme and followed the launch of the major UHC reform in Kenya. Overall, we found that low NHI enrolment prevailed among ISWs, with persistent pro-rich inequities, even among a population exclusively of ISWs. Our analysis further suggests that in addition to wealth, household size, educational status, one’s own employment sector, older age, MFI membership, and a positive experience with healthcare service delivery are all factors that shape enrolment. Hereafter, we appraise our findings in light of the existing literature and contextual factors, with the objective of proposing strategies that may promote higher enrolment rates among ISWs.

We note that after the COVID-19 pandemic, the overall NHI enrolment rate among ISWs remained low at 22%. While this represents an improvement from the 19% reported among ISWs in 2018 [42], eight out of ten ISWs in Kenya remain uninsured. This is a worrying trend, as Kenya has a large (84%) and fast-growing informal sector population [62, 63], whose enrolment in the NHI scheme, although mandated at the policy level, remains practically voluntary [36, 42]. Global evidence suggests that increasing NHI scheme enrolment through voluntary contributory mechanisms can be challenging [31, 55] unless specific strategies are put in place to motivate enrolment [64], which is not currently the case in Kenya. Previous studies in SSA have reported varied ISW enrolment rates in NHI schemes, ranging from 9.8% in Tanzania to 57% in Ghana [23–25, 65]. In Ghana, for example, the high NHI scheme enrolment rate, even among ISWs, has been attributed to robust bureaucratic frameworks, strong governance, innovative policy measures, public awareness campaigns, and innovative funding methods, all of which result in improved healthcare accessibility, affordability, and diverse equitable participation [66].

Our analysis draws attention to the fact that despite a slight increase in enrolment rates reported in our study compared with prior estimates, important pro-rich inequalities persist even when exclusively considering ISWs. The persistence of such inequalities is worrisome, as it undermines the pro-poor intention at the core of the Kenyan NHI schemes, as evidenced by its policies. The Kenyan government has long attempted to incorporate equity-oriented initiatives within the NHI scheme by subsidizing enrollment for low-income households. Subsidies have been an integral element of social health protection policies, including the National Health Insurance Subsidy Program (HISP), which was launched in 2014 and expanded in 2016 [67]; the transfer of free maternity policy from the Ministry of Health to the NHI scheme in 2016 [68]; and the post-Afya Care free healthcare pilot scale-up in 2021, which is currently established as the indigent program in the Social Health Insurance (SHI) Act of the 2023 [37, 67, 69]. These subsidies have been insufficient in the way that they are aimed at specific special groups, typically leaving the ISWs behind [33]. Expanding these subsidies to include ISWs who cannot afford premiums is crucial for improving NHI coverage rates among ISWs.

The poorest inequities, as captured by the concentration indices, were confirmed by the regression model results, suggesting that ISWs from the wealthiest quintile were more likely to enroll in the NHI scheme than those from the poorest quintile. This suggests that the NHI premium remains largely unaffordable for the poor, leaving them vulnerable to the economic consequences of health shocks. Our findings are consistent with those of previous studies conducted in LMICs, which reported pro-rich inequality in NHI enrollment despite the intention to be pro-poor. *Kazungu and Barasa* reported the existence of rich income-related inequalities in health insurance coverage in Kenya [55] Additionally, studies by *Jehu-Appiah *et al*., Akazili *et al*.,* and *Atakorah *et al*.* reported rich inequalities in enrolment [70], coverage [71] and subscription renewal [72], respectively, in the Ghana NHI scheme. While none of these studies specifically focused on ISWs, they provide a fair opportunity for comparison. The results from our regression analysis are consistent with the broader literature on demand for health insurance in LMICs, suggesting the existence of a relationship between wealth and the decision to enrol in a scheme [54, 55, 71, 73, 74]. Moreover, our regression analysis revealed that individuals from large households were less likely to enrol in the scheme. Since the NHI scheme premium does not increase with household size, our results can be explained by the household income distribution factor. In larger households, per capita income is often lower, stretching the available household income across other competing needs, making it difficult to prioritize health insurance premiums. Our findings are consistent with other studies, which report that larger households are less likely to enrol in health insurance schemes due to the associated financial burden [24, 55, 70, 75, 76].

Data emerging from the concentration curves in this analysis suggest that exposure to the ‘Afya Care’ free healthcare policy improved equity in NHI enrolment, but only for the poorest 20%, not among all socioeconomic quintiles. In the regression analysis, we observed a positive but nonsignificant association between exposure to the free healthcare policy and enrolment in the NHI, possibly confirming the pattern observed in the concentration curves. A plausible explanation for our findings is that during the consideration of post-Afya Care’s free healthcare policy scale-up, the Kenyan government targeted providing NHI premium subsidies for poor households, an initiative that started in the pilot counties before the policy was scaled up nationally [67]. Our findings corroborate evidence from previous studies conducted in low- and lower-middle-income countries. Subsidies for vulnerable populations are central to publicly funded health insurance schemes implemented in the SSA and are globally based on the principle of solidarity. In SSA, Ghana offers premium exemptions to vulnerable groups such as elderly individuals, children under 18, pregnant women, and livelihood program beneficiaries [28, 77, 78]; Rwanda’s 2004 policy subsidizes community-based health insurance coverage through formal sector contributions and donor funding [28, 79]; Gabon has expanded access to the poor by creating a dedicated fund [28, 80]; and Zambia’s NHI scheme aims to exempt social cash transfer beneficiaries from NHI scheme payments through a solidarity initiative [81, 82]. Globally, countries in Asian and Pacific regions, such as India, Indonesia, the Philippines, Thailand, Vietnam, Mongolia, and Cambodia, have effectively implemented various subsidized health insurance arrangements for vulnerable populations, including direct budget transfers, subsidized coverage for the informal sector and poor, and government subsidies [83].

Our findings also highlight how individuals employed in the non-agricultural sector are more likely to enrol than others. Considering that the effect also persists in the multivariate model adjusted for SES, we cannot attribute this association to the higher income individuals in the non-agricultural sector being more likely to enrol. In line with prior research [52, 53, 74], the effect is likely attributable to exposure to NHI concepts, proximity to NHI administrative structures, and residing in urban areas, elements that are more prevalent among ISWs holding positions outside the agricultural sector. Moreover, our analysis confirmed that individuals coming from households with MFI membership enjoyed a higher probability of enrolling in the NHI. Our findings are in line with previous evidence suggesting that MFIs can serve as intermediaries in promoting the enrolment of ISWs in emerging health insurance schemes [64, 84, 85], serving multiple functions such as increasing awareness, easing premium collection and payments, offering group enrolment options, and attracting relatively healthy individuals to the scheme, hence reducing adverse selection [10, 86–88]. These features make MFI a potential structure that can be leveraged as an onboarding structure within the informal sector. However, more context-specific studies are needed to assess the willingness, capacity, and structures required for effective implementation.

The positive association between prior healthcare experiences of the household members and individual decisions to enrol indicates that, not surprisingly, previous interactions with healthcare systems are fundamental in shaping the decision to invest resources in health insurance [89]. This finding highlights the underexplored behavioral and social spillover effect. We argue that a positive healthcare experience by one household member, regardless of whether they accessed it through the NHI scheme or not, may influence the perceptions of other household members. Positive encounters with healthcare services can foster trust in the system and enhance the perceived value of financial protection within intra-household discussions, potentially influencing enrollment choices. Consistent with our findings, various studies have linked higher health insurance enrolment and retention rates to positive healthcare experiences, whether through insured [33, 90–92] or uninsured [93] access by shaping trust, satisfaction, and perceived value. For instance, a randomized control trial in Ghana demonstrated that improving the quality of services increased insurance enrolment among initially uninsured households [93]. Individuals are willing to invest in health insurance only to the extent that they can trust the potential return on investment, which relies on the provision of good health services.

We note how higher household head education levels were associated with an increased likelihood of individual enrolment in the NHI scheme, which is consistent with findings from other LMICs such as Ghana, Burkina Faso, and Indonesia [24, 54, 56, 73, 74, 94]. A possible explanation for this finding is that individuals with low education levels may be less aware of the scheme. As such, they are likely to face administrative challenges in accessing information, navigating enrolment procedures, and understanding the benefit package, making enrolment difficult. The problem of access to information has been consistently reported in the literature on public health insurance, which threatens the effectiveness of public health insurance schemes, even when they are heavily subsidized [95–97]. Individuals who are unaware of their benefits cannot make use of them. Furthermore, our findings indicated that only one-third of the ISWs were highly educated, indicating the need for innovative communication and sensitization methods to reach the remaining two-thirds of low-educated ISWs. The use of community health volunteers (CHVs)^2^ as educators has been shown to improve health insurance coverage among low-income individuals [98]. In Kenya, CHVs have been widely and effectively utilized for other health messaging purposes. Policymakers in Kenya and elsewhere should explore and leverage the vast CHV network, which is already widely used for various health messaging purposes, to increase awareness of the NHI scheme.

Our results are also consistent with those of prior studies, indicating a higher probability of enrolling in health insurance among older individuals [26, 50, 52]. A plausible explanation is that people need more healthcare as they age to offset the increasing depreciation of health capital, thus choosing to purchase health insurance. This, however, could result in adverse selection and unsustainable pools. With slightly more than half of the informal sector workers between the ages of 18–34 years, the government should implement age-specific innovative strategies and incentives, such as digital applications that provide both education and enrolment, to motivate younger and healthier individuals to enrol in NHI schemes to balance the pool and cushion the elderly and sick. Moreover, to alleviate the financial burden on elderly individuals, one could consider full subsidization for older age groups, as is the case in countries such as Senegal [99] and Ghana [78, 90].

These findings highlight the need for targeted policy interventions that address the structural barriers to NHI enrolment among ISWs. Efforts should focus on improving affordability through subsidized premiums, enhancing literacy levels, and NHI awareness campaigns tailored to low-education populations through existing structures such as the community health strategy and microfinance institutions. Addressing the persistent pro-rich inequalities in enrollment is essential to ensure that countries move towards a mandatory NHI that does not exacerbate existing socioeconomic disparities, but rather promotes financial risk protection and equitable access to health coverage for all.

However, our findings should be interpreted in consideration of several limitations. First, the study is based on cross-sectional survey data, which limits our ability to make causal inferences. Our focus is on assessing the association between the outcome of interest, the enrolment in the NHI, and a series of sociodemographic and economic factors, without making any claims of causality. Secondly, while we acknowledge the value-added contribution of health service utilization information to this analysis, our dataset included service utilization questions in a limited way, as follow-up questions to specific illness episode responses, which limits generalizability to the entire sample. The few responses would affect the overall analytical power of this study, which focuses on enrollment, if included. We are, however, exploring how to utilize the data in a follow-up analysis. Thirdly, the survey is based on data collected in 2020. We recognize that the economic situation might have changed since then, in light of the increasing cost of living, inflation, shifting labour dynamics, and the long-term effects of the COVID-19 pandemic. However, considering the lack of more recent datasets on ISWs, the insights drawn from the 2020 data analysis and its implications remain highly relevant for understanding enrolment patterns and informing policymaking processes in Kenya. Additionally, some structural characteristics, such as the lack of formality in employment, and factors like trust in schemes, are likely to remain influential regardless of the overall economic situation. We, however, acknowledge that economic and policy changes could worsen inequalities in relation to the affordability of premiums. We therefore recommend the need for further research with more recent data to capture the correct status of the time-variant variables and their effect on NHIS enrolment. Furthermore, future studies should explore the retention levels or stability of ISWs in health insurance schemes, which are critical for measuring sustainability, as well as the extent to which insurance enables them to access care affordably.

## Conclusions

Our study investigated inequalities and factors associated with the decision to enrol in the NHI among ISWs in Kenya, a population segment that faces unique barriers to enrolment that have not been adequately addressed in the literature. Consistent with the low enrolment rate observed in our nationally representative sample and the persistent socioeconomic inequalities even among the population of ISWs, we postulate that new strategies are needed to increase participation and expand toward universal health coverage. Specifically, we recommend the use of nontraditional communication channels, such as CHWs and MFIs, to raise awareness among ISWs about the NHI scheme enrolment process and benefits packages and, where possible, to facilitate the enrolment process. More importantly, the government should consider a tax-based system, however, where not feasible, subsidies should be expanded to support the enrollment of other vulnerable groups beyond the ultra-poor, as too many competing needs limit available disposable income among communities living in the informal sector. However, further research is recommended to assess the feasibility, acceptability, and sustainability of these strategies in different settings. Our findings may also have applications beyond Kenya’s boundaries to other low-resource settings struggling to include ISWs in their NHI schemes.

## Data Availability

The datasets generated and/or analysed during the current study are available in the repository Dataset on Informal Employment, Social Security and Political Trust in six sub-Saharan African Countries / Friedrich-Ebert-Stiftung, German Institute of Development and Sustainability, International Labour Organisation.—(Version 1.0): Friedrich-Ebert-Stiftung. – 2023; https://tinyurl.com/3ptrvxjk [100].

## Abbreviations

AIC: Akaike’s information criterion
AOR: Adjusted odds ratio
CC: Concentration Curve
CHV: Community health volunteer
CI: Confidence interval
CIX: Concentration index
ICC: Intraclass correlation coefficient
ISWs: Informal sector workers
KES: Kenyan Shillings
LMICs: Low- and middle-income countries
MFIs: Micro Finance Institutions
NHI: National Health Insurance
PCV: Proportional change in variance
SES: Socioeconomic status
SSA: Sub-Saharan Africa
UHC: Universal Health Coverage
USD: United States Dollar
VIF: Variance Inflation Factor

## Footnotes

1 Informal Microfinance Institutions (MFIs) refer to rotational savings and credit associations (ROSCA) and accumulated savings and credit associations (ASCA), which enable members, mostly in the informal sector, to mobilize savings and access credit facilities [53].

2 Community Health Volunteers (CHVs) are individuals who are trained to deliver services in a defined geographical area location called a Community Health Unit by making home visits, providing health promotion messages, and treating common ailments and illnesses. CHVs operate within Kenya’s Community Health Strategy, which is part of the country’s broader health system aimed at achieving Universal Health Coverage (UHC).

## References

[1] United Nations. Goal 3: Ensure healthy lives and promote well-being for all at all ages [Internet]. 2016 [cited 2023 Dec 14]. Available from: https://www.un.org/sustainabledevelopment/health/

[2] Oxford Policy Management. Financing for Universal Health Coverage in low-and middle-income countries: a brief overview OPM seminar series on health financing for UHC Financing for Universal Health Coverage in low-and middle-income countries: a brief overview [Internet]. Oxford; 2016 Aug. Available from: www.opml.co.uk

[3] Agyepong IA, Abankwah DNY, Abroso A, Chun C, Dodoo JNO, Lee S, et al. The ‘universal’ in UHC and Ghana’s National Health Insurance Scheme: Policy and implementation challenges and dilemmas of a lower middle-income country. BMC Health Serv Res. 2016. 10.1186/s12913-016-1758-y. 27655007 10.1186/s12913-016-1758-y PMC5031274

[4] World Health Organization, International Bank for Reconstruction and Development / The World Bank. Tracking universal health coverage 2023 global monitoring report. Geneva; 2023.

[5] World Health Organization (WHO). Urgent health challenges for the next decade [Internet]. 2020 [cited 2023 Nov 30]. Available from: https://www.who.int/news-room/photo-story/photo-story-detail/urgent-health-challenges-for-the-next-decade?utm_source=STAT+Newsletters&utm_campaign=1931cb646b

[6] Advisory Board. The 13 biggest threats to global health, according to WHO [Internet]. https://www.advisory.com/daily-briefing/2020/01/15/who-health-challenges. 2023 [cited 2023 Nov 30]. Available from: https://www.advisory.com/daily-briefing/2020/01/15/who-health-challenges

[7] Tessema ZT, Worku MG, Tesema GA, Alamneh TS, Teshale AB, Yeshaw Y, et al. Determinants of accessing healthcare in sub-Saharan Africa: a mixed-effect analysis of recent demographic and health surveys from 36 countries. BMJ Open. 2022. 10.1136/bmjopen-2021-054397. 35105635 10.1136/bmjopen-2021-054397 PMC8804632

[8] Mulupi S, Kirigia D, Chuma J. Community perceptions of health insurance and their preferred design features: implications for the design of universal health coverage reforms in Kenya. BMC Health Serv Res [Internet]. 2013;13(1):474. 24219335 10.1186/1472-6963-13-474 PMC3842821

[9] Okungu V, Chuma J, Mulupi S, McIntyre D. Extending coverage to informal sector populations in Kenya: Design preferences and implications for financing policy. BMC Health Serv Res. 2018;18(1):13. 29316925 10.1186/s12913-017-2805-z PMC5761094

[10] Bonfert Anna, Özaltin Annette, Heymann Marilyn, Hussein Khizer, Langenbrunner Jack. Closing the Gap: Health Coverage for Non-Poor Informal-Sector Workers. 2015 Jul.

[11] Susan I, Justin Y, Rosalind P-R, Carol B. Health financing for universal health coverage in Sub-Saharan Africa: a systematic review. Glob Health Res Policy. 2021;6(1):8. 10.1186/s41256-021-00190-7. 33641673 10.1186/s41256-021-00190-7 PMC7916997

[12] Makochekanwa A. Informal Economy in SSA: Characteristics, size and tax potential [Internet]. 2020 Feb. Available from: https://mpra.ub.uni-muenchen.de/98644/

[13] Barasa E, Kazungu J, Nguhiu P, Ravishankar N. Examining the level and inequality in health insurance coverage in 36 sub-Saharan African countries. BMJ Glob Health. 2021. 10.1136/bmjgh-2020-004712. 33903176 10.1136/bmjgh-2020-004712 PMC8076950

[14] Prinja S, Chauhan AS, Karan A, Kaur G, Kumar R, Public Library of Science. Impact of publicly financed health insurance schemes on healthcare utilization and financial risk protection in India: a systematic review. PLoS ONE. 2017. 10.1371/journal.pone.0170996. 28151946 10.1371/journal.pone.0170996 PMC5289511

[15] National Department of Health. White Paper: Towards a national health insurance policy. [Internet]. Pretoria; 2017. Available from: www.gpwonline.co.za

[16] Nugraheni DA, Satibi S, Kristina SA, Puspandari DA. Factors associated with willingness to pay for cost-sharing under universal health coverage scheme in Yogyakarta, Indonesia: a cross-sectional survey. Int J Environ Res Public Health. 2022. 10.3390/ijerph192215017. 36429734 10.3390/ijerph192215017 PMC9690347

[17] Amoo BAG, Adenekan AT, Nagado HU. National Health Insurance Scheme (NHIS) implementation in Nigeria: issues, challenges, and way forward. CBN Bullion [Internet]. 2017;41:15–33.

[18] Muttaqien M, Setiyaningsih H, Aristianti V, Selby Coleman HL, Hidayat MS, Dhanalvin E, et al. Why did informal sector workers stop paying for health insurance in Indonesia? Exploring enrollees’ ability and willingness to pay. PLoS ONE. 2021;16:e0252708. 34086799 10.1371/journal.pone.0252708 PMC8177660

[19] Dartanto T, Halimatussadiah A, Rezki JF, Nurhasana R, Siregar CH, Bintara H, et al. Why do informal sector workers not pay the premium regularly? Evidence from the National Health Insurance System in Indonesia. Appl Health Econ Health Policy. 2020;18:81–96. 31535352 10.1007/s40258-019-00518-y

[20] Barasa EW, Mwaura N, Rogo K, Andrawes L. Extending voluntary health insurance to the informal sector: experiences and expectations of the informal sector in Kenya. Wellcome Open Res. 2017;2:94. 29387800 10.12688/wellcomeopenres.12656.1 PMC5698913

[21] Mbau R, Kabia E, Honda A, Hanson K, Barasa E. Examining purchasing reforms towards universal health coverage by the National Hospital Insurance Fund in Kenya. Int J Equity Health. 2020. 10.1186/s12939-019-1116-x. 32013955 10.1186/s12939-019-1116-x PMC6998279

[22] Social Health Insurance Fund (SHIF) review [Internet]. [cited 2025 Feb 4]. Available from: https://cytonn.com/topicals/social-health-insurance

[23] Adei D, Agyemang-Duah W, Mensah AA. Predictors of enrollment in a health protection scheme among informal sector workers in Kumasi Metropolis of Ghana. BMC Res Notes. 2019. 10.1186/s13104-019-4782-2. 31752971 10.1186/s13104-019-4782-2 PMC6873757

[24] Sekyi S, Domanban PB, Agbenyo F. Exploring heterogeneity of national health insurance scheme enrolment among persons in the informal sector. Int J Health Plann Manage. 2022;37:3282–96. 36002934 10.1002/hpm.3557

[25] Mwinuka B, Echoka E, Nyaberi JM. Uptake of health insurance and its associated factors among informal sector workers in Dar es Salaam, Tanzania. J Healthc Dev Countries. 2022;2:20–5.

[26] International Labour Laws (ILO). Actuarial analysis of the Zambia National Health Insurance Scheme and costing of the extension of coverage to Social Cash Transfer beneficiaries. Geneva; 2023.

[27] Nurhasana R, Hidayat B, Puspita Ratih S, Kusuma Hartono R, Dartanto T. The sustainability of premium payment of national health insurance’s self-enrolled members in Jakarta Greater Area. J Public Health Res. 2022;11(1):jphr-021. 10.4081/jphr.2021.2392 PMC8883551 34674517

[28] Cashin C, Dossou JP, Taylor and Francis Ltd. Can national health insurance pave the way to universal health coverage in sub-Saharan Africa? Health Syst Reform. 2021. 10.1080/23288604.2021.2006122. 34965364 10.1080/23288604.2021.2006122

[29] Delloite. Strategic Review of the National Hospital Insurance Fund-Kenya. 2011.

[30] Munyao Muiya B, Kamau A. Universal health care in Kenya: Opportunities and challenges for the informal sector workers. Int J Educ Res. 2013;1:1–10.

[31] Mathauer I, Schmidt JO, Wenyaa M. Extending social health insurance to the informal sector in Kenya. An assessment of factors affecting demand. Int J Health Plann Manage. 2008. 10.1002/hpm.914. 18050152 10.1002/hpm.914

[32] Schwettmann J. Covid-19 and the Informal Economy; Impact and Response Strategies in Sub-Saharan Africa. 2020 Aug.

[33] Bitran R. The world bank universal health coverage and the challenge of informal employment: Lessons from Developing Countries. Washington DC; 2014 Jan.

[34] Republic of Kenya. National Hospital Insurance Fund Act (revised 2012) [Internet]. 1998. Available from: www.kenyalaw.org

[35] Republic of Kenya. National Hospital Insurance Fund Act [Internet]. 2012. Available from: www.kenyalaw.org

[36] Ministry of Health. Kenya Universal Health Coverage Policy 2020__2030. 2020 [cited 2023 Aug 7]; Available from: https://www.health.go.ke/

[37] Shano Guyo, Ileana Vîlcu. A review of Afya Care – the Universal Health Coverage pilot program – in Isiolo county. Kenya Brief No.5 [Internet]. Washington, DC; 2020. Available from: https://thinkwell.global/

[38] Kiarie Josiah. Delivering quality and affordable health services: Kenya’s road to Universal Health Coverage (UHC) [Internet]. https://socialprotection.org/discover/blog/delivering-quality-and-affordable-health-services-kenya%E2%80%99s-road-universal-health#:~:text=Financing%20is%20the%20biggest%20hurdle%20facing%20the. 2022 [cited 2024 Mar 12]. Available from: https://socialprotection.org/discover/blog/delivering-quality-and-affordable-health-services-kenya%E2%80%99s-road-universal-health

[39] Republic of Kenya. The_NHIF_Amendment_Act_No.1_of_2022. 2022;

[40] Kenya National Bureau of Statistics. Kenya Demographic Health Survey. Ministry of Health [Internet]. 2022 [cited 2023 Sep 12];1. Available from: https://www.dhsprogram.com/pubs/pdf/FR380/FR380bis.pdf

[41] Kenya National Bureau of Statistics. Economic survey report. 2023.

[42] Ministry of Health. Kenya household health expenditure and utilization survey report. 2018 Jul.

[43] De La Rosa J, Scheil-Adlung X. Enabling transition to formalization through providing access to health care: The examples of Thailand and Ghana. ILO [Internet]. 2007. Available from: www.who.int/hdp/database

[44] Lee J, Di Ruggiero E. How does informal employment affect health and health equity? Emerging gaps in research from a scoping review and modified e-Delphi survey. Int J Equity Health. 2022. 10.1186/s12939-022-01684-7. 35725451 10.1186/s12939-022-01684-7 PMC9208971

[45] Kenya National Bureau of Statistics. 2019 Kenya Population and Housing Census: Volume II i. 2019.

[46] Kenya National Bureau of Statistics. Poverty report based on the 2019 Kenya continuous household survey. 2023.

[47] Di L, Katelyn G, Yoo J, Maina T. Staying ahead of the curve: challenges and opportunities for future spending on health in Kenya: Kenya Public Expenditure Review for the Health Sector - FY2014/15-FY2019/20. Washington DC; 2022 Jul.

[48] National Hospital Insurance Fund(NHIF). National Health Insurance Fund (NHIF) [Internet]. https://www.nhif.or.ke/schemes/. 2021 [cited 2024 Apr 2]. Available from: https://www.nhif.or.ke/schemes/

[49] ILO. Decent work and the informal economy [Internet]. Geneva; 2002. Available from: https://www.ilo.org/public/english/standards/relm/ilc/ilc90/pdf/rep-vi.pdf

[50] International Labour Laws (ILO). Effect to be given to resolutions adopted by the International Labour Conference at its 90th Session (2002), (b) Resolution concerning decent work and the informal economy; Governing Body, 285th Session, Seventh item on the agenda [Internet]. Geneva; 2000 Nov. Available from: https://www.ilo.org/sites/default/files/wcmsp5/groups/public/@dgreports/@integration/documents/publication/wcms_079142.pdf

[51] Strupat C. Social protection and social cohesion in times of the COVID-19 pandemic: evidence from Kenya. Eur J Dev Res. 2022. 10.1057/s41287-022-00541-1. 35578680 10.1057/s41287-022-00541-1 PMC9097141

[52] Amu H, Dickson KS, Kumi-Kyereme A, Maafo Darteh EK. Understanding variations in health insurance coverage in Ghana, Kenya, Nigeria, and Tanzania: evidence from demographic and health surveys. PLoS ONE. 2018;13:e0201833. 30080875 10.1371/journal.pone.0201833 PMC6078306

[53] Otieno PO, Wambiya EOA, Mohamed SF, Donfouet HPP, Mutua MK. Prevalence and factors associated with health insurance coverage in resource-poor urban settings in Nairobi, Kenya: a cross-sectional study. BMJ Open. 2019;9(12):e031543. 31843827 10.1136/bmjopen-2019-031543 PMC6924758

[54] Amu H, Dickson KS, Adde KS, Kissah-Korsah K, Darteh EKM, Kumi-Kyereme A. Prevalence and factors associated with health insurance coverage in urban sub-Saharan Africa: Multilevel analyses of demographic and health survey data. PLoS ONE. 2022;17(3):e0264162. 35245301 10.1371/journal.pone.0264162 PMC8896727

[55] Kazungu JS, Barasa EW. Examining levels, distribution and correlates of health insurance coverage in Kenya. Trop Med Int Health. 2017;22:1175–85. 28627085 10.1111/tmi.12912 PMC5599961

[56] Putri NK, Laksono AD, Rohmah N. Predictors of national health insurance membership among the poor with different education levels in Indonesia. BMC Public Health. 2023. 10.1186/s12889-023-15292-9. 36810024 10.1186/s12889-023-15292-9 PMC9945403

[57] Kabuya FI. The rotating savings and credit associations (ROSCAs): unregistered sources of credit in local communities. IOSR J Human Social Sci. 2015;20(8):95–8.

[58] O’Donnell O, van Doorslaer E, Wagstaff A, Lindelow M. Analyzing Health Equity Using Household Survey Data. Analyzing Health Equity Using Household Survey Data. The World Bank; 2007.

[59] World Health Organization. Handbook on health inequality monitoring: with a special focus on low- and middle-income countries [Internet]. WHO Library Cataloguing-in-Publication. WHO Library Cataloguing-in-Publication; 2013 [cited 2025 Jun 16]. Available from: https://www.who.int/publications/i/item/9789241548632

[60] Wagstaff A. The bounds of the concentration index when the variable of interest is binary, with an application to immunization inequality. Health Econ. 2005;14:429–32. 15495147 10.1002/hec.953

[61] Johnston R, Jones K, Manley D. Confounding and collinearity in regression analysis: a cautionary tale and an alternative procedure, illustrated by studies of British voting behaviour. Qual Quant. 2018;52:1957–76. 29937587 10.1007/s11135-017-0584-6 PMC5993839

[62] Murunga J, Muriithi MK, Wawire NW. Estimating the size of the informal sector in Kenya. Cogent Econ Finance. 2021. 10.1080/23322039.2021.2003000.

[63] Federation of Kenya Employers. > The Informal Economy in Kenya. 2021.

[64] Nigel J, Acharya Y. Increasing health insurance enrollment in low- and middle-income countries: what works, what does not, and research gaps: a scoping review. Inquiry (United States). 2022;59:00469580221090396. 10.1177/00469580221090396 PMC9121503 35574923

[65] Eric N-B, Moses A. Trends and characteristics of enrollment in the National Health Insurance Scheme in Ghana, a qualitative analysis of longitudinal data. Glob Health Res Policy. 2018;3(1):32. 30460332 10.1186/s41256-018-0087-6 PMC6233555

[66] Fusheini A, Marnoch G, Gray AM. Stakeholders perspectives on the success drivers in Ghana’s national health insurance scheme - identifying policy translation issues. Int J Health Policy Manag. 2017;6:273–83. 28812815 10.15171/ijhpm.2016.133 PMC5417149

[67] Maritim B, Nzinga J, Tsofa B, Musiega A, Mugo PM, Wong E, et al. Evaluating the effectiveness of the National Health Insurance Subsidy Programme within Kenya’s universal health coverage initiative: a study protocol. BMJ Open. 2024;14:e083971. 39578024 10.1136/bmjopen-2024-083971 PMC11590815

[68] Orangi S, Kairu A, Ondera J, Mbuthia B, Koduah A, Oyugi B, et al. Examining the implementation of the Linda Mama free maternity program in Kenya. Int J Health Plann Manage. 2021;36:2277–96. 34382238 10.1002/hpm.3298 PMC9290784

[69] Government of Kenya. Social Health Insurance Act 16 of 2023. Laws of Kenya; Nov, 2023.

[70] Jehu-Appiah C, Aryeetey G, Spaan E, de Hoop T, Agyepong I, Baltussen R. Equity aspects of the National Health Insurance Scheme in Ghana: who is enrolling, who is not and why? Soc Sci Med. 2011;72:157–65. 21145152 10.1016/j.socscimed.2010.10.025

[71] Akazili J, Welaga P, Bawah A, Achana FS, Oduro A, Awoonor-Williams JK, et al. Is Ghana’s pro-poor health insurance scheme really for the poor? Evidence from Northern Ghana. BMC Health Serv Res. 2014. 10.1186/s12913-014-0637-7. 25494816 10.1186/s12913-014-0637-7 PMC4268792

[72] Atakorah YB, Arthur E, Osei-Fosu AK, Novignon J. Economic inequalities in health insurance subscription renewal: evidence from Ghana’s National Health Insurance Scheme. Soc Sci Med. 2024;341:116514. 38142607 10.1016/j.socscimed.2023.116514

[73] Dake FAA. Examining equity in health insurance coverage: an analysis of Ghana’s National Health Insurance Scheme. Int J Equity Health. 2018. 10.1186/s12939-018-0793-1. 29914497 10.1186/s12939-018-0793-1 PMC6006705

[74] Parmar D, De Allegri M, Savadogo G, Sauerborn R. Do community-based health insurance schemes fulfil the promise of equity? A study from Burkina Faso. Health Policy Plan. 2014;29:76–84. 23307908 10.1093/heapol/czs136

[75] De Allegri M, Sanon M, Bridges J, Sauerborn R. Understanding consumers’ preferences and decision to enrol in community-based health insurance in rural West Africa. Health Policy. 2006;76:58–71. 15946762 10.1016/j.healthpol.2005.04.010

[76] Ozawa S, Grewal S, Bridges JFP. Household size and the decision to purchase health insurance in Cambodia: results of a discrete-choice experiment with scale adjustment. Appl Health Econ Health Policy. 2016;14:195–204. 26860280 10.1007/s40258-016-0222-9 PMC4791455

[77] Dixon J, Tenkorang EY, Luginaah I. Ghana’s national health insurance scheme: a national level investigation of members’ perceptions of service provision. BMC Int Health Hum Rights. 2013. 10.1186/1472-698X-13-35. 23968385 10.1186/1472-698X-13-35 PMC3765400

[78] Kanchebe Derbile E, Van Der Geest S. Repackaging exemptions under National Health Insurance in Ghana: how can access to care for the poor be improved? Health Policy Plan. 2013;28:586–95. 23065542 10.1093/heapol/czs098

[79] Chemouni B. The political path to universal health coverage: power, ideas and community-based health insurance in Rwanda. World Dev. 2018;106:87–98.

[80] Mibindzou Mouelet Ange, El Idrissi Moulay, Robyn Paul Jacob. Gabon indigents scheme a social health insurance program for the poor. Universal Health Coverage Study Series No. 31. 2018.

[81] Osei Afriyie D, Titi-Ofei R, Masiye F, Chansa C, Fink G. The political economy of national health insurance schemes: evidence from Zambia. Health Policy Plan [Internet]. 2024;40(1):66–74. 10.1093/heapol/czae094/7822267). 10.1093/heapol/czae094 PMC11725516 39404000

[82] Zombe M, Mowowo Eners C, Chilufya Kalaba M, Malan M. The National Health Insurance Scheme (NHIS) and the Attainment of Universal Health Coverage in Zambia. Rwanda Public Health Bull. 2023;4(1):53–6.

[83] Vilcu I, Probst L, Dorjsuren B, Mathauer I. Subsidized health insurance coverage of people in the informal sector and vulnerable population groups: trends in institutional design in Asia. Int J Equity Health. 2016;15:1–29. 27716301 10.1186/s12939-016-0436-3 PMC5050723

[84] International Labour Laws (ILO). Extension of social health insurance to workers in the informal economy through organized groups. 2015.

[85] Banerjee A, Duflo E, Hornbeck R. Bundling health insurance and microfinance in India: there cannot be adverse selection if there is no demand. Am Econ Rev Am Econ Assoc. 2014;5:291–7. 10.1257/aer.104.5.291 PMC4159356 25214652

[86] Churchill C. Protecting the poor - A microinsurance compendium; microinsurance operation 3. 2006.

[87] Leatherman S, Geissler K, Gray B, Gash M. Health financing: a new role for microfinance institutions? J Int Dev. 2013;25:881–96.

[88] Orange Eloise, Andre Glenn. PlaNet Paper Innovation at the heart of health microinsurance schemes in Africa. Paris; 2013.

[89] Markowitz W, Kausar K, Coffield E. Relationship between patient experience scores and health insurance. Healthcare. 2022. 10.3390/healthcare10112128. 36360469 10.3390/healthcare10112128 PMC9690600

[90] Delavallade C. Quality health care and willingness to pay for health insurance retention: a randomized experiment in kolkata slums. Health Econ (United Kingdom). 2017;26:619–38. 10.1002/hec.3337 27028701

[91] Acharya D, Raikhola PS, Subedi KR, Devkota B, Bhattarai R, Pathak KP, et al. Qualitative evaluation of the health insurance program in Nepal: expectations beyond limitations. World Med Health Policy. 2024;16:37–56.

[92] Gurung GB, Panza A. Predictors of annual membership renewal to increase the sustainability of the Nepal National Health Insurance program: a cross-sectional survey. PLoS Glob Public Health. 2022. 10.1371/journal.pgph.0000201. 36962197 10.1371/journal.pgph.0000201 PMC10021716

[93] Duku SK, Nketiah-Amponsah E, Fenenga CJ, Janssens W, Pradhan M. The effect of community engagement on healthcare utilization and health insurance enrollment in Ghana: Results from a randomized experiment. Health Econ. 2022;31(10):2120. 35944042 10.1002/hec.4556 PMC9545140

[94] Sundays ME. Determinants of uptake and utilization of national hospital insurance fund medical cover by people in the informal sector in Kakamega County Kenya. Univers J Public Health. 2015;3:169–76.

[95] Parisi D, Srivastava S, Parmar D, Strupat C, Brenner S, Walsh C, et al. Awareness of India’s national health insurance scheme (PM-JAY): a cross-sectional study across six states. Health Policy Plan. 2023;38:289–300. 36478057 10.1093/heapol/czac106 PMC10019566

[96] Dror DM, Shahed Hossain SA, Majumdar A, Koehlmoos TLP, John D, Panda PK. What factors affect voluntary uptake of community-based health insurance schemes in low- and middle-income countries? A systematic review and meta-analysis. PLoS One: Public Library of Science; 2016. 10.1371/journal.pone.0160479 PMC5006971 27579731

[97] Platteau J-P, Ontiveros DU. Understanding and Information Failures: Lessons From a Health Microinsurance Program in India. 2013 Feb. Report No.: 29.

[98] ShresthaVaidya Marina, Manandhar Naresh, Dhimal Meghnath, Joshi Kumar Sunil. View of Awareness on Social Health Insurance Scheme among Locals in Bhaktapur Municipality. Journal of BP Koirala Institute of Health Sciences. 2021;4. 10.33314/jnhrc.v18i3.2471 33210634

[99] Local Health System Sustainability Project (LHSS) under Integrated Health Systems IDIQ. Expanding Financial Protection by Addressing Non-financial Barriers: Senegal Case Study [Internet]. Rockville; 2022 Jun. Available from: https://p4h.world/en/countries/senegal/

[100] Dataset on Informal Employment, Social Security and Political Trust in six sub-Saharan African Countries / Friedrich-Ebert-Stiftung, German Institute of Development and Sustainability, International Labour Organisation. - (Version 1.0) : Friedrich-Ebert-Stiftung. - 2023. - Dataset. [Internet]. 2023 [cited 2025 Jan 17]. Available from: https://www.fes.de/en/africa-department/a-majority-working-in-the-shadows?amp%3Btx_form_formframework%5Bcontroller%5D=FormFrontend&tx_form_formframework%5Baction%5D=perform&cHash=7656af7defb171b50198d181ad5165eb

